# Interferon‐γ Mitigates Oxaliplatin‐Induced Neurotoxicity: Analogies With Connexin 43

**DOI:** 10.1002/cns.71197

**Published:** 2026-10-22

**Authors:** Alessandra Toti, Valentina Ferrara, Elena Lucarini, Laura Micheli, Clara Ciampi, Francesco Margiotta, Daniel Venturi, Carmen Parisio, Elisabetta Gerace, Lorenzo Curti, Alessio Masi, Tommaso Mello, Guido Mannaioni, Carla Ghelardini, Lorenzo Di Cesare Mannelli

**Affiliations:** ^1^ Department of Neuroscience, Psychology, Drug Research and Child Health—NEUROFARBA—Pharmacology and Toxicology Section University of Florence Florence Italy; ^2^ European Biomedical Research Institute of Salerno (EBRIS) Salerno Italy; ^3^ Department of Health Science University of Florence Florence Italy; ^4^ Clinical Gastroenterology Unit, Department of Biomedical Clinical and Experimental Sciences “Mario Serio” University of Florence Florence Italy

**Keywords:** hemichannels, IFN‐γ dualism, neuropathy, neurotrophic factors, pro‐inflammatory cytokine, spinal cord

## Abstract

**Aims:**

Chemotherapy‐induced neuropathy is a major side effect of platinum‐based drugs such as oxaliplatin. Given the context‐dependent pro‐inflammatory or neuroprotective actions of interferon‐γ (IFN‐γ), this study investigated its role in an in vitro model of oxaliplatin‐induced neurotoxicity (OIPN).

**Methods:**

Rat organotypic spinal cord slices were exposed to oxaliplatin to model chemotherapy‐induced neurotoxicity responses in vitro. IFN‐γ cytotoxicity was assessed at 6 and 24 h. Neurotoxicity, inflammatory mediators, neurotrophic factors, and markers of glial and neuronal activation were analyzed by biochemical and immunohistochemical assays. Connexin 43 (Cx43) was assessed using Gap19 and Gap27 to probe hemichannel and gap‐junction contributions. Electrophysiological recordings characterized neuronal excitability.

**Results:**

IFN‐γ (6.5–200 ng/mL) was non‐toxic and, at 65 ng/mL, significantly reduced oxaliplatin‐induced neurotoxicity, decreasing LDH release and suppressing pro‐inflammatory mediators (CCL2, SerpinA3N, CGRP, substance P, c‐Fos). IFN‐γ modulated its signaling by reducing IFNγR and increasing IRF1 expression. It prevented oxaliplatin‐induced overexpression of neurotrophic factors and reduced glial and neuronal hyperactivation markers, preserving neuronal integrity. IFN‐γ reduced Cx43 expression and hemichannel activity, and Gap19 reproduced selected protective effects. Electrophysiology showed attenuation of oxaliplatin‐induced neuronal hyperexcitability.

**Conclusion:**

IFN‐γ attenuates oxaliplatin‐induced neurotoxic responses in an in vitro spinal cord model. The parallel reduction in Cx43 expression and hemichannel activity supports further investigation of Cx43 signaling as a candidate pharmacological target in OIPN.

## Practitioner Points

IFN‐γ reduces oxaliplatin‐induced neurotoxicity and preserves neuronal integrity in organotypic spinal cord slices.

IFN‐γ suppresses pro‐inflammatory mediators and hyperactivation markers in spinal cord slices.

Connexin 43 hemichannel modulation is associated with the neuroprotective profile of IFN‐γ.

## Graphical abstract

IFN‐γ mitigates oxaliplatin‐induced neurotoxicity by reducing neuroinflammation and neuronal excitability, suggesting a protective mechanism involving Cx43 modulation and potential therapeutic value in chemotherapy‐induced neurotoxicity.

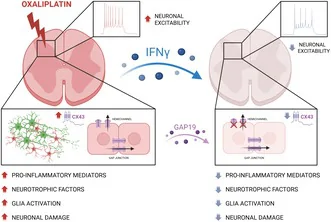

## 1 Introduction

Neurotoxicity is a common adverse effect of various chemotherapeutic agents, metabolic disorders, and inflammatory conditions, leading to structural and functional damage of the peripheral and central nervous systems [1]. Among antineoplastic drugs, oxaliplatin, a third‐generation platinum compound, is widely used for its strong antitumor efficacy [2] but is frequently limited by oxaliplatin‐induced peripheral neurotoxicity (OIPN), which affects 30%–40% of treated patients [3]. The severity and persistence of OIPN depend on cumulative dose, infusion parameters, and individual susceptibility factors, including pre‐existing pathological conditions such as diabetes [4]. Although its pathophysiology remains incompletely understood, proposed mechanisms include ion channel dysregulation [5], mitochondrial dysfunction with oxidative stress [6] and glial‐driven neuroinflammation [7].

Glial cells play a pivotal role in neurotoxicity. Under physiological conditions, they support neuronal metabolism and synaptic transmission; however, following injury or toxic insult, they undergo reactive gliosis, shifting toward a pro‐inflammatory phenotype [8]. In the spinal cord, a critical relay center for sensory integration and pain transmission, damage‐associated molecular patterns (DAMPs) activate local immune responses, prompting microglia and astrocytes to release cytokines such as interleukin (IL)‐6, IL‐1β, tumor necrosis factor‐α (TNF‐α), and interferon‐γ (IFN‐γ), along with other pronociceptive mediators [9]. While this response can exacerbate neuroinflammation and neuronal hyperexcitability, glia also contribute to tissue protection by clearing apoptotic cells and promoting resolution of inflammation [10].

Among the inflammatory mediators, IFN‐γ stands out for its pleiotropic and context‐dependent actions in the central nervous system. Depending on the concentration, cellular context, and molecular targets, IFN‐γ may act as either neurotoxic or neuroprotective [11, 12, 13, 14]. After peripheral nerve injury, elevated IFN‐γ is released by activated astrocytes and damaged neurons [11]. Its pro‐nociceptive effects are well established: in the dorsal horn, IFN‐γ facilitates neuron–glia communication critical for neuropathic pain development [15], enhances excitatory synaptic activity, downregulates AMPA receptors on inhibitory interneurons, and impairs GABAergic transmission, thereby disrupting the excitatory/inhibitory balance [16]. Furthermore, IFN‐γ decreases Connexin 43 (Cx43) expression through JNK activation, weakening gap junction‐mediated coupling and further contributing to neuronal hyperexcitability [17].

Conversely, IFN‐γ can also trigger neuroprotective pathways [18]. It promotes the release of brain‐derived neurotrophic factor (BDNF), supporting neuronal survival, plasticity, and cortical neurogenesis [19]. During acute neuroinflammation, IFN‐γ–induced IL‐6 release from astrocytes exerts anti‐apoptotic effects [20], while in chronic autoimmune conditions, IFN‐γ activates the immunoproteasome, enhancing the clearance of misfolded proteins and reducing oxidative stress [18, 21, 22].

Astrocytes are key targets of IFN‐γ signaling. Although prolonged or excessive exposure to IFN‐γ drives them toward a neurotoxic phenotype, characterized by reduced glutamate uptake and increased excitotoxicity [13, 14], low levels of IFN‐γ upregulate excitatory amino acid transporters (EAATs), enhancing glutamate clearance and maintaining synaptic homeostasis [13]. Noteworthy, recent transcriptomic studies performed in neuropathic mice further revealed downregulation of IFN‐γ signaling in parabrachial astrocytes, suggesting that impaired astrocytic responsiveness may heighten vulnerability to pain [23, 24]. However, despite growing evidence linking IFN‐γ to both neuroinflammatory and neuroprotective processes, its precise role in oxaliplatin‐induced peripheral neurotoxicity, as well as the cellular and molecular mechanisms underlying its dual actions within the spinal cord, remain to be elucidated.

In this context, IFN‐γ emerges as a multifaceted modulator of glial function and neuronal excitability, capable of exerting both protective and deleterious effects depending on the neuroinflammatory context [18]. This study investigates its dual role in the spinal cord, using organotypic slice cultures as an in vitro model of oxaliplatin‐induced neurotoxicity, with the goal of clarifying how IFN‐γ influences neuronal excitability and exploring pharmacologically relevant pathways.

## 2 Materials and Methods

### 2.1 Study Approval

All animal procedures complied with Directive 2010/63/EU, IASP recommendations, and the Guide for the Care and Use of Laboratory Animals (NIH). Experiments were approved by the Italian Ministry of Health (No. 17.E9C.N.XCN) and the Animal Subjects Review Board of the University of Florence, and were reported in accordance with ARRIVE guidelines [25]. All efforts were made to minimize animal suffering and reduce animal use.

### 2.2 Preparation of Rat Organotypic Spinal Cord Slice Cultures

Organotypic spinal cord slices were prepared from postnatal Day 5–6 (P5–P6) Sprague Dawley rat pups of both sexes, as previously described [26]. Following preparation, slices were maintained under standard culture conditions (37°C, humidified atmosphere containing 5% CO_2_) for 14 days in vitro before experimental treatments.

### 2.3 Organotypic Spinal Cord Cultures Treatments

On the 14^th^ day in vitro, organotypic spinal cord culture was exposed to: oxaliplatin (10 μM); IFN‐γ (6.5–200 ng/mL); Gap19 and Gap27 (10–200 μM) at different timings, depending on experimental condition. The IFN‐γ concentrations were selected to encompass a broad range of biologically active concentrations previously used in spinal astrocyte and dorsal horn neuronal cultures [16, 17], corresponding approximately to 30, 300, and 1000 U/mL based on the biological activity of the recombinant rat IFN‐γ preparation used.

### 2.4 Cytotoxicity Assay

Cytotoxicity was assessed by measuring lactate dehydrogenase (LDH) release into the culture medium using a Cytotoxicity Detection Kit (Roche, Mannheim, Germany), according to the manufacturer's instructions. Each value corresponded to one well (*n* = 6 slices). LDH levels were normalized to total protein content measured by BCA assay (Merck, Milan, Italy). Data are expressed as percentage of untreated controls, set at 100%.

### 2.5 RNA Isolation, Reverse Transcription and RT‐PCR

Total RNA was isolated from rat organotypic spinal cord slices using TRI Reagent (Merck, Milan, Italy). 500 ng of RNA was retrotranscribed using PrimeScript RT reagent Kit with gDNA eraser. RT‐PCR was performed using SsoAdvanced Universal SYBR Green Supermix following the thermal profile suggested by the kit. The primers used are reported in Table 1 (Invitrogen, Monza, Italy). The following validated primers were purchased from Bio‐Rad (Milan, Italy): rIL‐1a (qRnoCID0002952), rIL‐1b (qRnoCID0004680), rTac1 (qRnoCID0007480), rCalca (qRnoCED0004252), rFos (qRnoCED0002247), rScl1a2 (qRnoCED0005967), rIfngr1 (qRnoCID0001888), rIrf1 (qRnoCID0003410), rBdnf (qRnoCED0005012), rGdnf (qRnoCED0007415), rTgfb1 (qRnoCID0009191), rNefh (qRnoCED0002393), rMapk1 (qRnoCID0003206), rGja1 (qRnoCED0006203), rβ2m (qRnoCED0056999) and rGapdh (qRnoCID0057018). The differential expression of the transcripts is normalized on the housekeeping genes 18S, Gapdh and β2m.

**TABLE 1:** List of forward and reverse primers used for RT‐PCR.

Gene	Forward	Reverse
rCCL2	5′‐TCTTCCTCCACCACTATGCAGGTCTC‐3′	5′‐TCTTTGGGACACCTGCTGCTGGTG‐3′
rSerpinA3N	5′‐CTTTCTGCAGTATGTGGGAATCACTTGG‐3′	5′‐GGCTGCATTGCTCTAAGTAGGAGTGC‐3′
rSCL1A3	5′‐CAGTCATCGTCGGCCTCCTCATTC‐3′	5′‐CTGGTGATGCGTTTGTCCACACCATTG‐3′
rVEGFA	5′‐AGACTCTTCGAGGAGCACTTTGGGTC‐3′	5′‐ATGTGTGTGTATGTGGGTGGGTGTGT‐3′
rGFAP	5′‐CTGACACACGTTGTGTTCAAGCAGCC‐3′	5′‐CTGAAGGTTAGCAGAGTTGACAAGGG‐3′
rS100B	5′‐TCAGGGAGAGAGGGTGACAAGCAC‐3′	5′‐GGCTGTGGTCACCATGGAGACGAAG‐3′
rMBP	5′‐GCAGCTTCCGAAGGCCTGGATGT‐3′	5′‐GCCAGAGCCCCGCTTGGGCGCAC‐3′
18S	5′ GGGGAATCAGGGTTCGATTCCG‐3′	5′ GGCACCAGACTTGCCCTCCAATG‐3′

*Note:* The sequences listed in this table refer to primers specifically designed for this study.

### 2.6 Immunofluorescence Staining in Free‐Floating

Organotypic spinal cord slice cultures were fixed and processed for immunofluorescence as previously described [26], with the following modifications. After fixation with cold 4% paraformaldehyde at 4°C overnight, slices were blocked in TBS containing 0.3% Triton X‐100 and 10% normal goat serum. Slices were incubated overnight at 4°C with primary antibody chicken anti‐GFAP (1:1000; Life Technologies, Monza, Italy), followed by incubation with Alexa Fluor 647 goat anti‐chicken secondary antibody (1:1000; Life Technologies, Monza, Italy). Neuronal nuclei were labeled using Alexa Fluor 488–conjugated anti‐NeuN antibody (1:200; Millipore, Milan, Italy). Nuclei were counterstained with DAPI, and slices were mounted using Fluoromount prior to imaging.

Images were acquired using a motorized Leica DM6000 B fluorescence microscope. GFAP and NeuN fluorescence intensity was quantified from two fields per slice (8–9 slices total) using a 20× objective, while NeuN^+^ cell counts were obtained from four fields per slice (9 slices total) using a 40× objective.

### 2.7 Western Blot Analysis

At Day 14 in vitro, treated organotypic spinal cord slices were collected and lysed in ice‐cold lysis buffer containing protease inhibitors (Roche, Milan, Italy). Lysates were sonicated, centrifuged, and supernatants were collected. Protein concentration was determined using a BCA assay (Merck, Milan, Italy). Equal amounts of protein (20 μg) were separated by SDS–PAGE and transferred to nitrocellulose membranes (Bio‐Rad, Milan, Italy). Membranes were blocked and incubated overnight at 4°C with primary antibodies against Connexin 43 (rabbit anti‐Cx43, 1:8000; Merck, Milan, Italy) and α‐tubulin (mouse anti‐α‐tubulin, 1:5000; Merck, Milan, Italy). After incubation with HRP‐conjugated secondary antibodies (Bethyl, Montgomery, TX, USA; Santa Cruz Biotechnology, Dallas, TX, USA), immunoreactive bands were visualized by enhanced chemiluminescence (Pierce ECL, Rockford, IL, USA). Densitometric analysis was performed using ImageJ software (NIH, Bethesda, MD, USA). Cx43 levels were normalized to α‐tubulin and expressed as a percentage of control values.

### 2.8 EtBr Uptake and Confocal Imaging

For dye uptake experiments, organotypic spinal cord cultures were incubated with ethidium bromide (EtBr, 5 μM; Merck, Milan, Italy) at 37°C. To evaluate the contribution of connexin hemichannels, slices were pre‐incubated with Gap19 (10 μM) for 30 min prior to EtBr exposure. After EtBr incubation, slices were washed in artificial cerebrospinal fluid, fixed with cold 4% paraformaldehyde at 4°C overnight, and processed for immunofluorescence as described above, with the following modifications.

Slices were incubated with mouse anti‐S100β antibody (1:400; Merck, Milan, Italy) to identify astrocytes, followed by Alexa Fluor 488–conjugated goat anti‐mouse secondary antibody (1:1000; Life Technologies, Milan, Italy). Nuclei were counterstained with DAPI and sections were mounted using Fluoromount. Confocal images were acquired using a Leica SP8 confocal microscope with a HC PL APO CS2 63×/1.40 oil‐immersion objective. Z‐stacks (6 μm thickness, 0.3 μm step size) were collected in sequential mode (excitation: 488 nm for S100β and 580 nm for EtBr). Image analysis was performed using ImageJ software. EtBr uptake was quantified in S100β^+^ cells as the ratio between EtBr fluorescence intensity and nuclear area.

### 2.9 Electrophysiological Recording on Somatosensory Neurons in the Dorsal Horn

Whole‐cell patch clamp recordings were performed in the dorsal horn of organotypic spinal cord slices. Individual slices were placed in a recording chamber and perfused with warm (33°C–34°C), carbo‐oxygenated artificial cerebrospinal fluid (aCSF; 95% O_2_/5% CO_2_) containing (in mM): 130 NaCl, 3.5 KCl, 1.25 NaH_2_PO₄, 24 NaHCO₃, 10 glucose, 2 CaCl_2_, and 1 MgSO₄. Patch pipettes (3–4 MΩ) were pulled from thin‐walled borosilicate glass (Harvard Apparatus) and filled with a K^+^‐gluconate–based intracellular solution (in mM): K^+^‐gluconate, 15 KCl, 10 HEPES, 0.1 EGTA, 2 MgCl_2_, 5 Na_2_‐phosphocreatine, 0.3 NaGTP, and 2 MgATP (pH 7.25–7.3; 280–300 mOsm). Slices were visualized using an upright infrared microscope (Nikon Eclipse E600FN), and dorsal horn neurons were identified based on their anatomical location relative to the ventral fissure [27]. Passive membrane properties were recorded using Clampex 10 software. Intrinsic excitability was assessed by current–voltage (I–V) relationships obtained from depolarizing current steps (−50 to +225 pA, 25 pA increments). Resting membrane potential and action potential threshold were determined in current‐clamp mode. Local synaptic connectivity was evaluated by recording spontaneous excitatory postsynaptic currents (sEPSCs) at a holding potential of −70 mV, analyzing frequency, amplitude, rise time, decay time, and area. Signals were acquired at 10 kHz, low‐pass filtered at 3 kHz using an Axon Multiclamp 700B amplifier (Molecular Devices, Sunnyvale, CA, USA), and analyzed offline with pCLAMP and GraphPad Prism software.

### 2.10 Biochemical Analysis

At the end of treatments, culture media were collected for biochemical analysis. CGRP and substance P levels were quantified by ELISA using commercial kits (BIOMATIK, EKF58049; MyBioSource, MBS703659), according to the manufacturers' instructions. Values were normalized to total protein content measured by BCA assay (Merck, Milan, Italy) and expressed as a percentage of untreated controls, set at 100%.

### 2.11 Statistics

Data are presented as mean ± SEM. Statistical analysis was performed using one‐way ANOVA with Bonferroni post hoc test. Electrophysiological data were analyzed by two‐way ANOVA followed by Tukey's multiple comparison test, while action potential parameters were assessed using Sidak's post hoc test. A *p* value < 0.05 was considered statistically significant. Analyses were performed using Origin 9.1 software.

## 3 Results

### 3.1 Neuroprotective Role of IFN‐γ in Oxaliplatin‐Induced Toxicity

LDH release assay was employed to assess potential IFN‐γ–associated toxicity and identify an effective concentration. Organotypic spinal cord slices were treated with different concentrations of IFN‐γ (6.5, 65, or 200 ng/mL) for 6 or 24 h, and none of these treatments affected cell viability (Figure S1A,B). To induce neurotoxic damage, slices were exposed to oxaliplatin (OXA, 10 μM) for the same experimental times, based on our previous work [26], resulting in a 61% and 96% increase in LDH release, respectively (Figure 1A,B).

**FIGURE 1:**
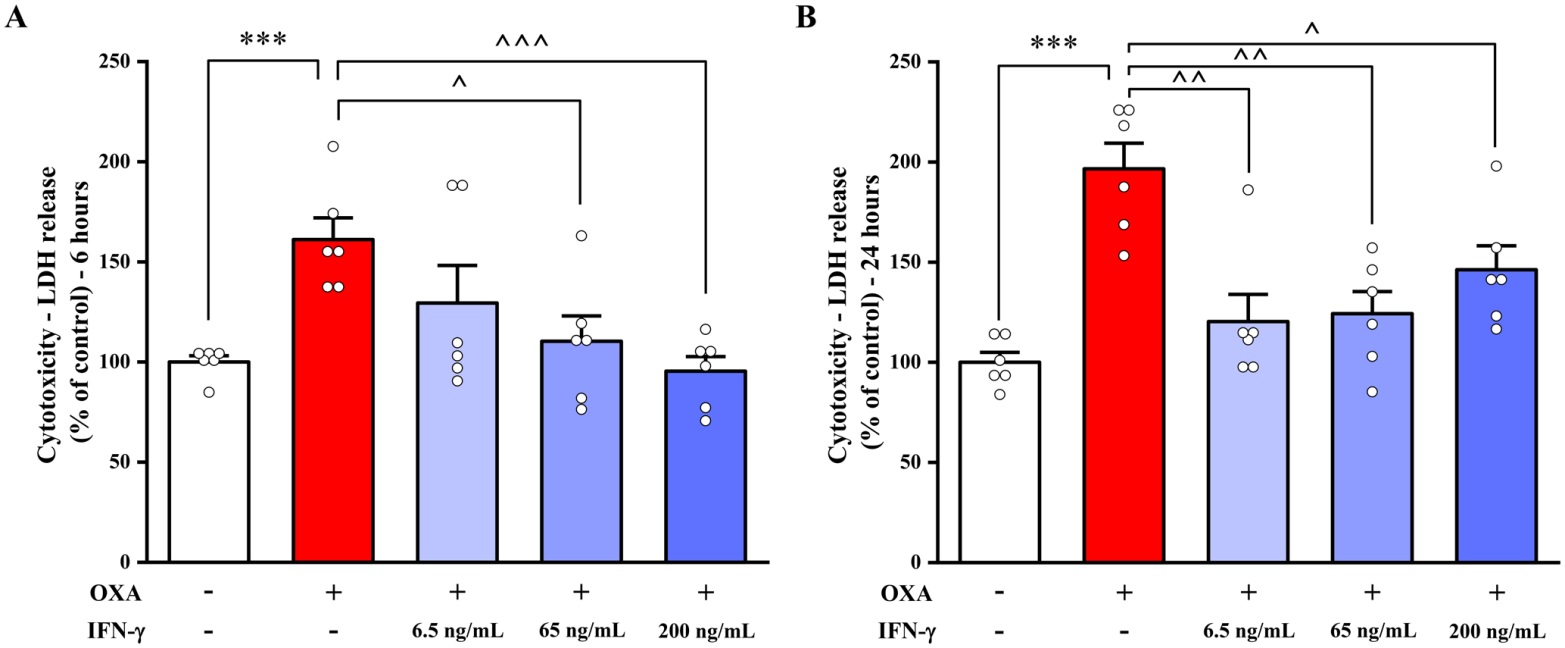
Cytotoxicity assessment in organotypic spinal cord slices treated with oxaliplatin and IFN‐γ. LDH quantification in the culture medium after 6 (A) and 24 h (B) of treatment with oxaliplatin (OXA, 10 μM) in combination with three different concentrations of IFN‐γ (6.5, 65, and 200 ng/mL). Results are expressed as mean ± SEM (*n* = 6). Values are expressed as a percentage of the control, arbitrarily set at 100. ***p < 0.001 vs control; ^^^ *p* < 0.05; ^^^^ *p* < 0.01; ^^^^^ *p* < 0.001 vs oxaliplatin.

Co‐treatment with IFN‐γ significantly reduced oxaliplatin‐induced damage. After 6 h, toxicity was attenuated by 30% at the lowest IFN‐γ dose and 65% at the highest, though the maximal 65% reduction fell to 50% after 24 h. Notably, 65 ng/mL of IFN‐γ provided significant and sustained protection at both time points (110.3 ± 12.7 and 124.3 ± 11.1, respectively), prompting its selection for subsequent experiments.

### 3.2 Impact of Co‐Treatment With IFN‐γ and Oxaliplatin on Gene Expression Profile in Organotypic Spinal Cord Slices

The expression of inflammation‐, nociception‐, cell activation‐, and neurotrophic‐related genes was investigated by RT‐PCR in organotypic spinal cord slices treated for 6 h with oxaliplatin (10 μM), IFN‐γ (65 ng/mL), and their combination. As shown in the heatmap (Figure 2), oxaliplatin induced an upregulation of Il1b (4.48 ± 0.82), Ccl2 (7.71 ± 0.77), Serpina3n (3.20 ± 0.64), Tac1 (Substance P, SP, 2.0 ± 0.34), and Fos (c‐FOS, 2.05 ± 0.25) mRNA expression. Co‐treatment with IFN‐γ significantly reduced the expression of Ccl2 (2.11 ± 0.62) and Fos (0.70 ± 0.07), while a decreasing trend was observed for Tac1 (1.05 ± 0.10) and Il1b (3.41 ± 0.30). Notably, co‐treatment also led to a reduction in Calca mRNA levels (Calcitonin gene‐related peptide, CGRP, 0.47 ± 0.08). Additionally, ELISA analysis conducted on the media confirmed a significant increase in the neurotransmitters SP and CGRP after 3 and 6 h of oxaliplatin treatment, followed by a tendency toward reduction upon IFN‐γ co‐treatment (Figure S2A,B). Co‐treatment with oxaliplatin and IFN‐γ enhanced the expression of Slc1a3 (1.60 ± 0.15) and Slc1a2 (1.91 ± 0.21), which encode the excitatory amino acid transporters (EAAT) 1 and EAAT2, respectively, compared to control levels. Notably, Slc1a2 expression was also significantly higher in the co‐treatment condition than with oxaliplatin alone (0.91 ± 0.24).

**FIGURE 2:**
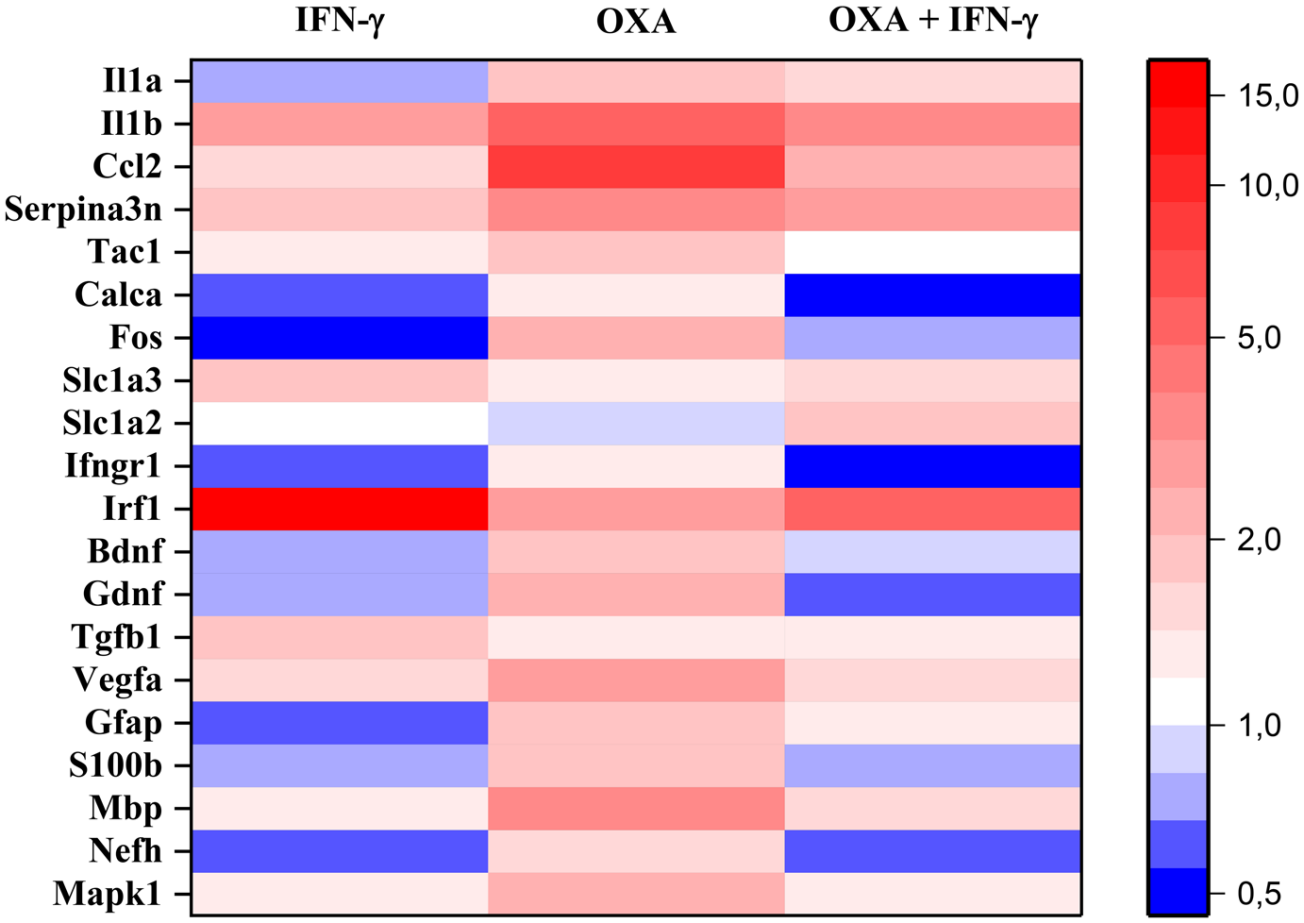
Heatmap of gene expression related to inflammation, nociception, cell activation, and neurotrophic/neuroprotective factors. Organotypic spinal cord slices treated with oxaliplatin (OXA, 10 μM) and IFN‐γ (65 ng/mL), alone or in combination. Results are expressed as fold changes in comparison to control (arbitrarily set to 1) and correspondence of colors and fold changes were reported on the right side of the figure. mRNA levels are expressed as the mean of three independent experiments. The analysis of variance was performed by one‐way ANOVA test and Bonferroni's significant difference procedure was used as a post hoc comparison.

Regarding IFN‐γ signaling, IFN‐γ treatment reduced Ifngr1 (IFN‐γ receptor 1) mRNA expression by approximately 40% (0.61 ± 0.09) compared to the control. Additionally, co‐treatment effectively counteracted the oxaliplatin‐induced increase of Ifngr1 expression (0.45 ± 0.1 vs. 1.32 ± 0.24). Interestingly, Irf1, a gene whose transcription is driven by IFN‐γ‐dependent STAT1 phosphorylation and nuclear translocation [28], exhibited a seventeen‐fold increase under IFN‐γ treatment (17.51 ± 1.16) compared to the control. However, the presence of oxaliplatin attenuated this induction by nearly six‐fold (5.78 ± 0.56), suggesting a distinct regulatory response.

Oxaliplatin treatment promoted the upregulation of neurotrophic and neuroprotective factors, including Bdnf (1.65 ± 0.07), Gdnf (2.15 ± 0.18), and Vegfa (2.54 ± 0.15). Co‐treatment with IFN‐γ effectively normalized Bdnf (0.87 ± 0.12) and Gdnf (0.64 ± 0.19) expression, while Vegfa expression is reduced by nearly 50% (1.35 ± 0.17). Oxaliplatin significantly increased the mRNA expression of key markers of cellular activation, including S100b (2.01 ± 0.05), Mbp (3.46 ± 0.64), Nefh (Nf‐H, 1.62 ± 0.12) and Mapk1 (2.12 ± 0.08). Co‐treatment with IFN‐γ strongly reduced these activation markers, restoring their expression to control levels. Notably, IFN‐γ alone decreased Nefh expression by approximately 40% (0.59 ± 0.05). Additionally, Gfap expression was examined, showing a tendency to increase following oxaliplatin treatment and to decrease upon co‐treatment.

### 3.3 Mitigation of Oxaliplatin‐Induced Cellular Activation and Neuronal Distress Through IFN‐γ Co‐Treatment

The sustained reduction in cellular activation was validated by immunofluorescence analysis of GFAP and NeuN after 24 h of treatment.

As shown in Figure 3A,B oxaliplatin increased GFAP fluorescence intensity three‐fold compared to control, while IFN‐γ co‐treatment substantially decreased GFAP fluorescence intensity by over 35% compared to oxaliplatin‐treated slices. Moreover, oxaliplatin reduced NeuN fluorescence intensity by more than 50% and decreased NeuN^+^ cells by 40% in comparison to control (Figure 3A,C,D). Notably, co‐treatment with IFN‐γ slightly alleviates the reduction in NeuN fluorescence but significantly prevents a~40% decline in NeuN^+^ cell numbers in comparison with oxaliplatin treatment.

**FIGURE 3:**
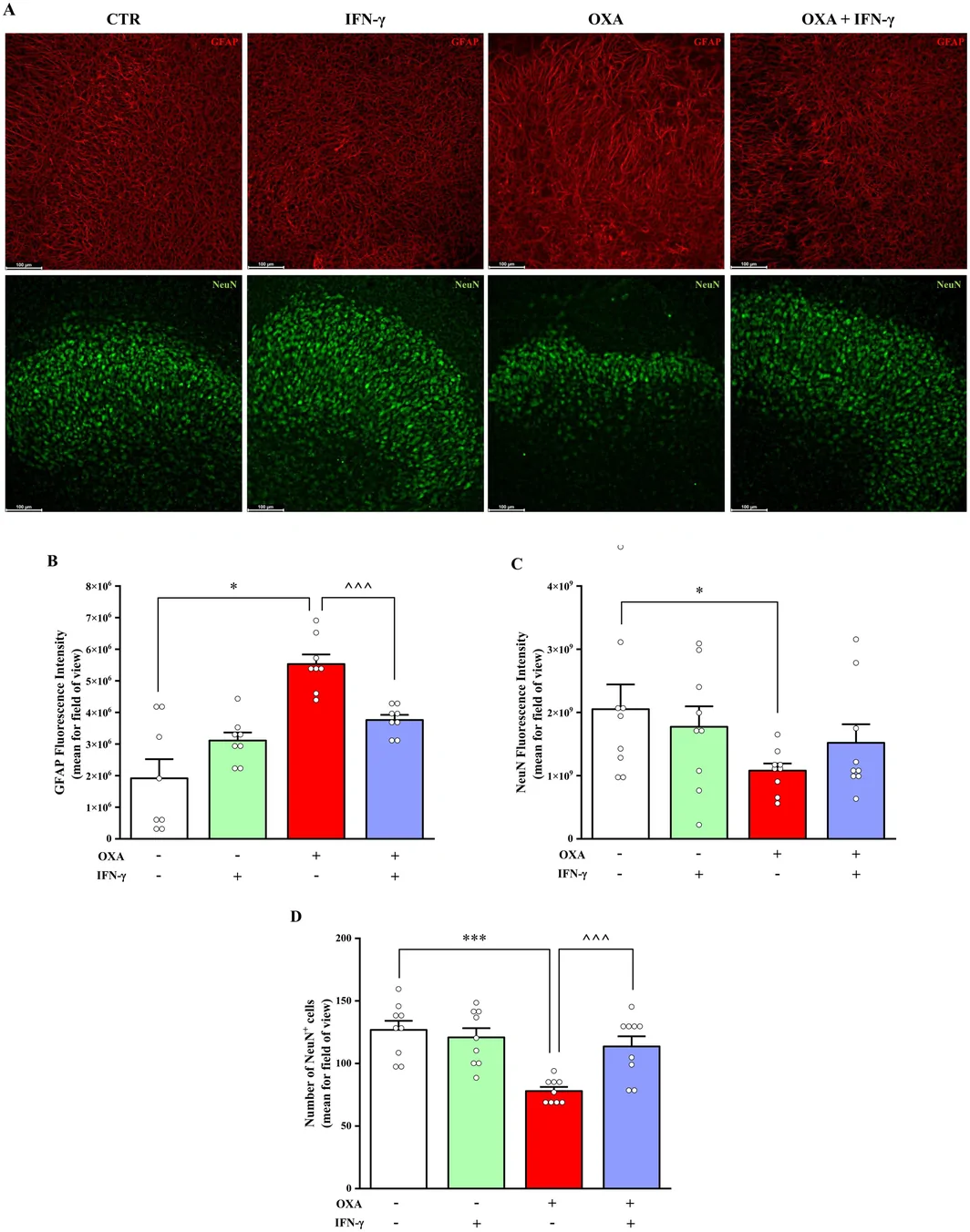
Analysis of morphological alterations in glial and neuronal cells. (A) Representative immunofluorescence image of GFAP (in red) and NeuN (in green) conducted on organotypic spinal cord slices treated for 24 h with oxaliplatin (OXA, 10 μM) and IFN‐γ (65 ng/mL), both as single treatments and in co‐treatment. Scale bar = 100 μm, 20× objective. (B) GFAP Fluorescence Intensity; (C) NeuN Fluorescence Intensity; (D) Number of NeuN^+^ cells. Results are expressed as mean ± SEM of three independent experiments with *n* = 8 slices for GFAP and *n* = 9 slices for NeuN analysis. *p < 0.05; ***p < 0.001 vs. control; ^^^^^ *p* < 0.001 vs. oxaliplatin.

### 3.4 Connexin 43 Channel Modulation Parallels IFN‐γ‐Dependent Neuroprotection: Differential Hemichannel and Gap‐Junction Effects

Cx43 has been implicated as a potential mediator of IFN‐γ–dependent neuroprotection [29]. Therefore, we first assessed whether IFN‐γ modulates Cx43 expression in oxaliplatin‐treated organotypic spinal cord slices. After 6 h of treatment, oxaliplatin significantly increased GJA1 mRNA expression (~2‐fold), an effect that was fully prevented by IFN‐γ co‐treatment (Figure 4A). To investigate whether IFN‐γ selectively influences Cx43 function through hemichannels (HCs) or gap junctions (GJs), organotypic slices were pre‐incubated with the Cx43‐selective inhibitors Gap19 (HC blocker) or Gap27 (GJ blocker) before oxaliplatin exposure [30, 31]. After 6 h, both inhibitors significantly reduced oxaliplatin‐induced cytotoxicity in a concentration‐dependent manner (Figure 4B). However, at 24 h, high concentrations of both peptides exacerbated cytotoxicity, an effect particularly evident with Gap27, whereas Gap19 at 10 μM did not increase LDH release (Figure 4C). These findings suggest distinct and time‐dependent roles of Cx43 HCs and GJs in oxaliplatin‐induced neurotoxicity.

**FIGURE 4:**
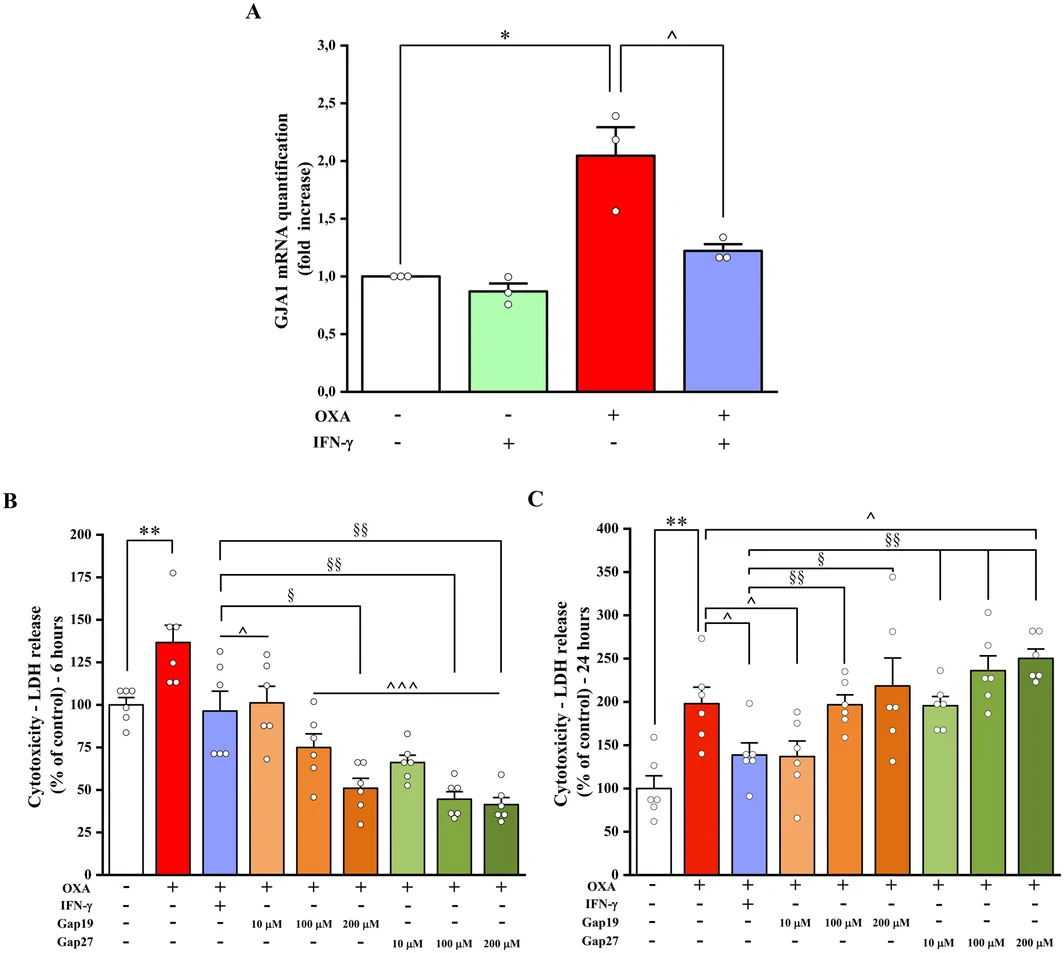
Modulation of Cx43 expression and LDH release by IFN‐γ in oxaliplatin‐treated organotypic spinal cord slices. (A) Cx43 (GJA1) mRNA expression assessed by RT‐PCR after 6 h treatment with oxaliplatin (OXA, 10 μM) or IFN‐γ (65 ng/mL), alone or in combination. (B–C) Cytotoxicity assessment by LDH release in the culture medium following partial or concentration‐dependent inhibition of Cx43 hemichannels (Gap19) or gap junctions (Gap27) after 6 h (B) and 24 h (C) of co‐treatment with oxaliplatin (10 μM). Results are expressed as mean ± SEM. RT‐PCR data derive from three independent experiments, while LDH data represent *n* = 6 samples. LDH values are expressed as percentages relative to untreated controls, arbitrarily set at 100%. *p < 0.05, **p < 0.01 vs. control; ^^^ *p* < 0.05, ^^^^^ *p* < 0.001 vs. oxaliplatin; ^§^ *p* < 0.05, ^§§^ *p* < 0.01 vs. oxaliplatin + IFN‐γ.

### 3.5 Convergent Effect of IFN‐γ and Gap19 on Cx43 Expression and Hemichannel Activity in Oxaliplatin‐Induced Neurotoxicity

To corroborate the transcriptional findings and determine whether IFN‐γ and Gap19 also modulate Cx43 at the protein level, GJA1 mRNA and Cx43 protein expression were quantified by RT‐PCR and Western blot in organotypic spinal cord slices treated with oxaliplatin (10 μM) and/or IFN‐γ (65 ng/mL), or preincubated for 30 min with the selective hemichannel inhibitor Gap19 (10 μM). Oxaliplatin‐induced upregulation of GJA1 mRNA was paralleled by a~55% increase in Cx43 protein levels (Figure 5B,C). Notably, IFN‐γ alone significantly reduced Cx43 protein expression despite unchanged mRNA levels, suggesting post‐transcriptional regulation.

**FIGURE 5:**
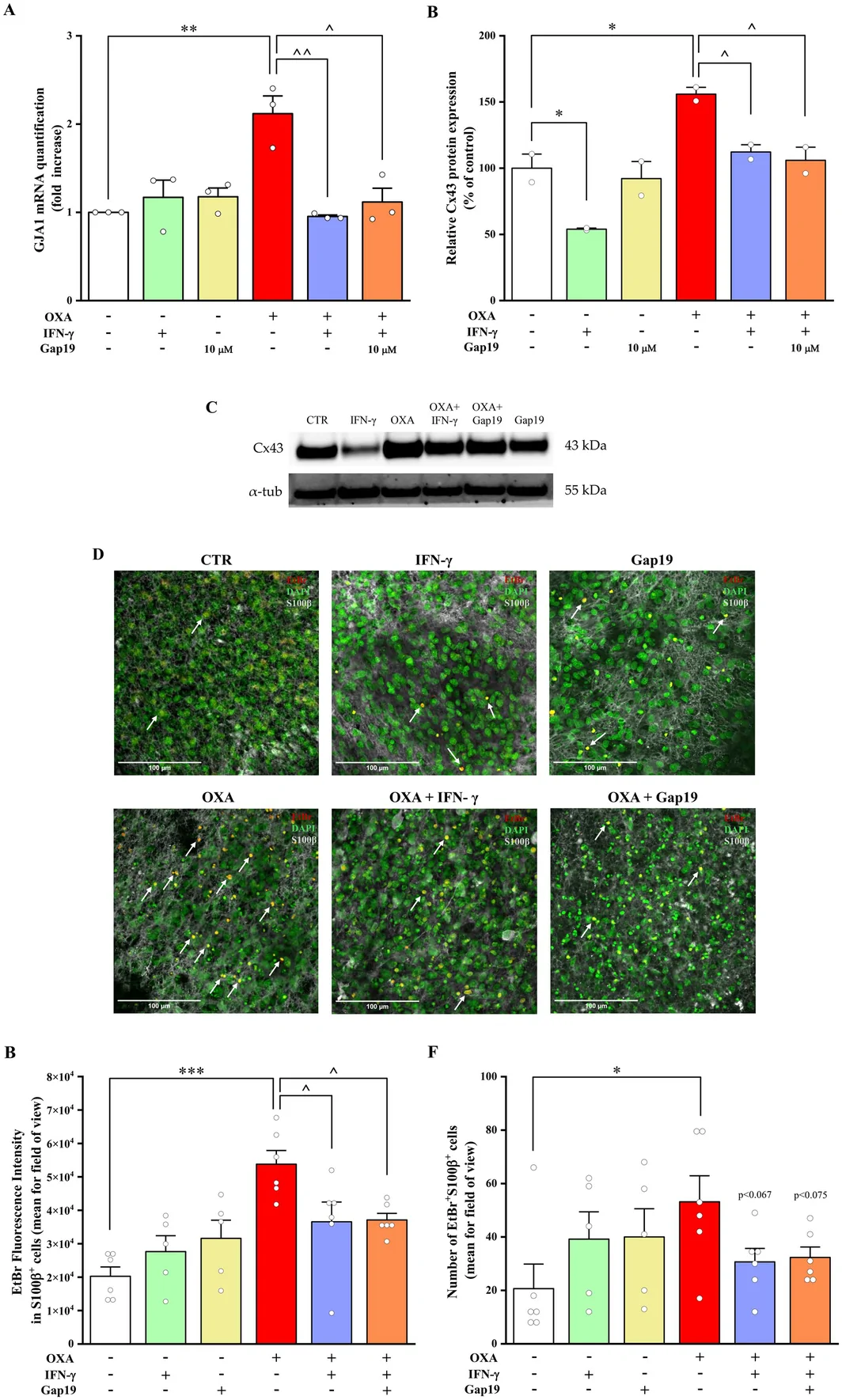
Effects of IFN‐γ and Gap19 on Cx43 expression and hemichannel functionality in oxaliplatin‐treated organotypic spinal cord slices. (A, B) Cx43 (GJA1) mRNA (A) and protein (B) expression levels evaluated after treatment with oxaliplatin (OXA, 10 μM) and IFN‐γ (65 ng/mL), as well as following a 30‐min pre‐treatment with the selective Cx43 hemichannel inhibitor Gap19 (10 μM), administered alone or in combination. (C) Representative Western blot image of Cx43 protein expression. (D–F) Assessment of Cx43 hemichannel functionality by ethidium bromide (EtBr, 5 μM) uptake after 24 h treatment with oxaliplatin (10 μM), IFN‐γ (65 ng/mL), or following 30‐min pre‐treatment with Gap19 (10 μM). (D) Representative confocal images showing DAPI (green), S100β (gray), and EtBr (red). (E) Quantification of EtBr fluorescence intensity. (F) Percentage of S100β^+^ astrocytes displaying EtBr uptake. Scale bar = 100 μm; 63× objective. Data are expressed as mean ± SEM. Gene and protein expression data derive from at least two independent experiments (*n* = 2), while EtBr uptake data derive from three independent experiments with *n* = 5–6 slices. *p < 0.05; **p < 0.01; ***p < 0.001 vs. control; ^^^ *p* < 0.05; ^^^^ *p* < 0.01 vs. oxaliplatin.

Preincubation with Gap19 alone did not affect GJA1 mRNA expression; however, in oxaliplatin‐treated slices, Gap19 significantly reduced both Cx43 mRNA and protein levels to values comparable to those observed under IFN‐γ co‐treatment (Figure 5A–C).

Cx43 hemichannel (HC) activity was next assessed by measuring ethidium bromide (EtBr) uptake in organotypic slices treated with oxaliplatin and/or IFN‐γ for 24 h. Astrocytes, identified by S100β immunolabeling [32], were selected as the reference population due to their high Cx43 expression [29]. Oxaliplatin markedly increased EtBr uptake in S100β^+^ astrocytes (~2.5‐fold), whereas co‐treatment with IFN‐γ or Gap19 reduced EtBr fluorescence intensity by approximately 65% and decreased the proportion of EtBr^+^ astrocytes (Figure 5D–F).

### 3.6 Neuromodulatory Effects of IFN‐γ on Somatosensory Neurons in the Dorsal Horn of Organotypic Spinal Cord Slices

To investigate the pathophysiological mechanisms underlying chemotherapeutic neurotoxicity, electrophysiological responses of somatosensory neurons in the dorsal horn of organotypic spinal cord slices were analyzed following 2–4 h exposure to oxaliplatin (10 μM), IFN‐γ (65 ng/mL), and/or Gap19 (10 μM), alone or in combination. Membrane capacitance, membrane resistance, and resting membrane potential were not affected by any treatment (Figure S3A–C).

Oxaliplatin significantly enhanced intrinsic neuronal excitability, with a pronounced effect in the 150–200 pA range (Figure 6A). Co‐treatment with IFN‐γ partially attenuated neuronal hyperexcitability, although less effectively than a 30‐min preincubation with Gap19, likely reflecting the higher selectivity of Gap19 for Cx43 hemichannels and the shorter IFN‐γ exposure. Neuronal firing threshold was not altered by any treatment (Figure S3D).

**FIGURE 6:**
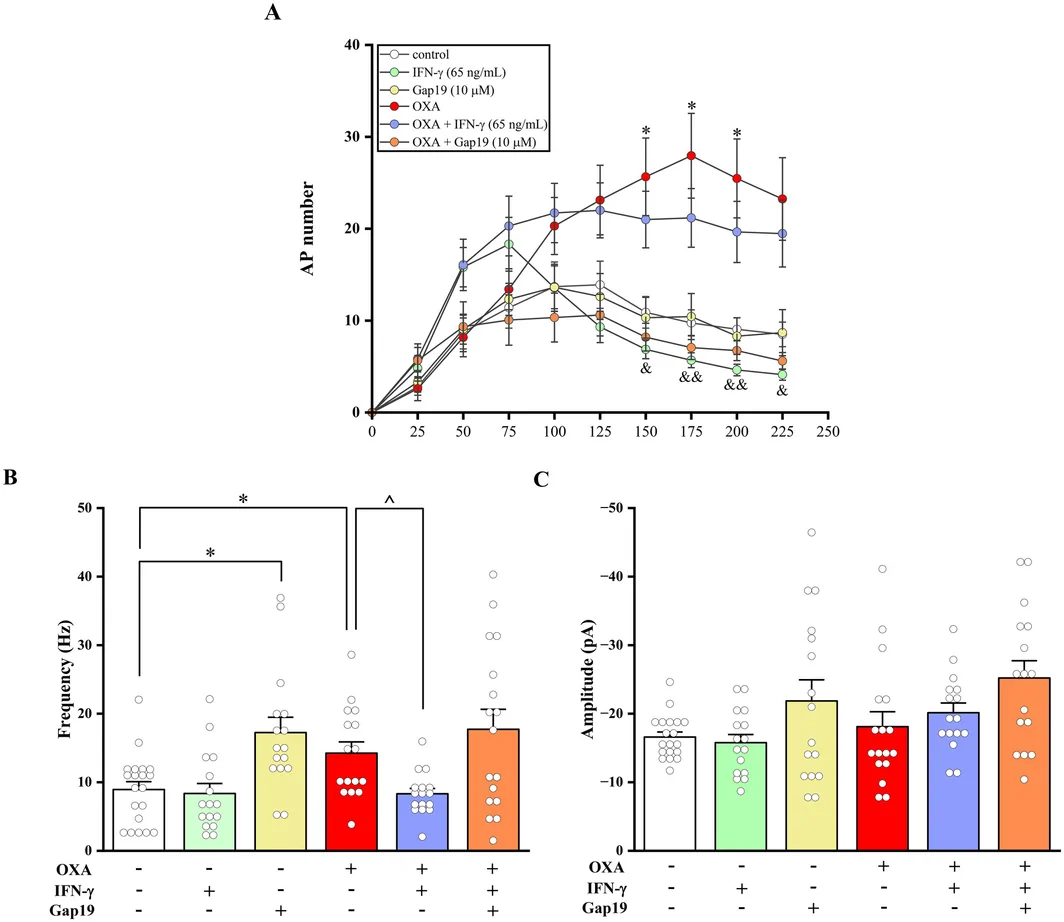
Electrophysiological effects of IFN‐γ on oxaliplatin‐induced neuronal hyperexcitability and synaptic activity in dorsal horn neurons. (A) Total number of action potentials (APs) recorded from somatosensory neurons in the dorsal horn of organotypic spinal cord slices after 24 h treatment with oxaliplatin (OXA, 10 μM), IFN‐γ (65 ng/mL), or their combination. Gap19 (10 μM) was applied as a 30‐min pretreatment, either alone or together with OXA. (B–C) Analysis of local neuronal connectivity assessed by whole‐cell voltage‐clamp recordings of spontaneous excitatory postsynaptic currents (sEPSCs). (B) Frequency (Hz) and (C) amplitude (pA) of sEPSCs measured under the same experimental conditions described in (A). Data are expressed as mean ± SEM and derive from at least 15 cells for AP analysis and 16 cells for sEPSC recordings, collected from three independent experiments. *p < 0.05 vs. control; ^*p* < 0.05 vs. oxaliplatin; ^&^ *p* < 0.05, ^&&^ *p* < 0.01 oxaliplatin + Gap19 vs. oxaliplatin.

In parallel, slices co‐treated with oxaliplatin and IFN‐γ exhibited a marked reduction in spontaneous excitatory postsynaptic current (sEPSC) frequency compared to oxaliplatin alone (Figure 6B). A trend toward increased sEPSC amplitude was also observed following IFN‐γ or Gap19 co‐treatment (Figure 6C), while no significant changes were detected in sEPSC kinetic properties (Figure S4).

## 4 Discussion

Our current research highlights the complex yet promising neuroprotective role of IFN‐γ, a cytokine traditionally recognized for its pro‐inflammatory properties, in the context of oxaliplatin‐induced neurotoxicity. Building on existing literature addressing the dual role of IFN‐γ in pain modulation [11, 15, 18, 20, 33] and supported by our previous transcriptomic analysis [23, 24], which identified alterations in regulatory pathways including IFN‐γ signaling in neuropathic pain, we sought to investigate the functional consequences of IFN‐γ in chemotherapy‐induced neurotoxicity.

Produced by both immune and neural cell types, IFN‐γ exerts complex context‐dependent effects: sustained or excessive signaling in a pro‐inflammatory environment may disrupt the excitatory‐inhibitory balance, impair astrocytic glutamate handling, exacerbate neuroinflammation, and promote mechanical allodynia [13, 14, 15, 16, 34], whereas more limited stimulation may activate neuroprotective responses, including glial‐derived BDNF secretion [19] and IL‐6‐mediated anti‐apoptotic signaling [20]. These divergent effects are influenced by multiple factors, including cytokine concentration, duration of exposure, cellular targets, and the pathological environment in which IFN‐γ signaling occurs.

To investigate these properties, we utilized organotypic spinal cord slices, which, although they do not reproduce the systemic and peripheral components of OIPN, preserve native cytoarchitecture and local neuron–glia interactions, enabling controlled evaluation of cellular responses and bridging in vitro and in vivo models [26].

Consistent with prior evidence of IFN‐γ–mediated neuronal protection in toxic injury paradigms [27], we observed that IFN‐γ (6.5–200 ng/mL) attenuated oxaliplatin‐induced cytotoxicity. The protective profile varied according to concentration and exposure time: 200 ng/mL produced the greatest protection at 6 h, but its effect was less pronounced at 24 h, whereas 65 ng/mL provided significant protection at both time points and was therefore selected for subsequent experiments.

Oxaliplatin exposure is known to activate intracellular pathways that drive cellular stress and neuroinflammatory responses, exacerbating pro‐nociceptive signaling [35, 36]. In our study, IFN‐γ suppressed oxaliplatin‐induced production of inflammatory and pro‐algic mediators including CCL2, SerpinA3N, substance P, and reduced c‐Fos induction. CCL2 is a key chemokine that enhances spinal nociceptive transmission and sensitization via CCR2‐dependent mechanisms [37]. In parallel, the reduced activation marker c‐Fos is relevant because c‐Fos‐dependent transcriptional programs contribute to neuroinflammatory amplification and pain‐related plasticity. Overall, this anti‐inflammatory/pro‐resolving profile is consistent with the concept that IFN‐γ can exert protective actions depending on timing, concentration, and cellular context [11, 15, 18].

Importantly, IFN‐γ downregulated glial activation markers GFAP and S100β, both associated with reactive phenotypes [38, 39]. While S100β at low levels supports homeostasis, its upregulation can promote neuroinflammation and maladaptive signaling [40]. In addition, oxaliplatin increased MAPK1 expression, a kinase involved in pain sensitization and hyperalgesia [41], and elevated markers linked to structural stress responses. Co‐treatment with IFN‐γ reduced these activation signatures and supported neuronal integrity, consistent with its capacity to promote neuronal survival and differentiation programs [19]. These findings strengthen the idea that IFN‐γ can shift the glial response away from a maladaptive state, limiting secondary neuronal distress.

Neurotrophic factors such as BDNF, GDNF, and VEGF‐A support neuronal survival; their overproduction contributes to neuronal hyperexcitability and pain hypersensitivity [42, 43, 44]. Consistent with this, our findings confirm that oxaliplatin increases these factors, while IFN‐γ co‐treatment reduces their levels, suggesting suppression of hyperexcitability mechanisms.

Supporting IFN‐γ's anti‐nociceptive role, we observed a reduction in CGRP and SP expression, two key mediators of dorsal horn pain processing [45]. CGRP receptor binding sites are enriched in superficial dorsal horn circuits, where CGRP signaling facilitates excitatory transmitter release and amplifies nociceptive processing [46]. Thus, the attenuation of CGRP/SP induced by IFN‐γ suggests a mechanism through which neuroimmune modulation may translate into reduced pro‐algic output.

IFN‐γ has also been implicated in the regulation of neuronal excitability in pain pathways. Prior work indicates that IFN‐γ can induce features of central sensitization in dorsal horn neurons under pro‐inflammatory conditions [16]. Intriguingly, our findings reveal that, in the context of oxaliplatin exposure, IFN‐γ instead exerts a protective neuromodulatory profile: electrophysiological recordings confirmed that oxaliplatin increased intrinsic excitability, while IFN‐γ co‐treatment tended to mitigate this hyperexcitability and specifically reduced oxaliplatin‐induced increases in sEPSC frequency. Together, these results suggest that IFN‐γ limits chemotherapy‐triggered neuronal hyperactivity, consistent with the broader neuromodulatory capacity of inflammatory cytokines in shaping excitability depending on state and circuit context [47].

Emerging evidence indicates that IFN‐γ modulates glial–neuronal communication via Cx43 [17], a gap junction and hemichannel protein expressed in astrocytes [29] and other cell types and implicated in neuroinflammatory signaling. In neuropathic states, Cx43 contributes to gliotransmitter release, ATP efflux, and central sensitization [48]. Consistent with this view, our data show that oxaliplatin exposure upregulated Cx43 expression and hemichannel activity, as indicated by enhanced EtBr uptake, whereas IFN‐γ co‐treatment downregulated Cx43 and reduced hemichannel functionality.

Pharmacological dissection further supported a differential contribution of Cx43 configurations. Using Gap 19, a selective hemichannel inhibitor [30, 49] and Gap27, a gap junction inhibitor, applied at concentrations commonly reported in the literature for organotypic and primary culture models [50, 51], we observed an early reduction in LDH release with both agents across all tested concentrations, consistent with the protective effect of partial Cx43 inhibition [49]. However, prolonged Gap27 exposure increased cytotoxicity, indicating that broad gap junction blockade may disrupt essential intercellular homeostasis. In contrast, low‐dose Gap19 produced effects partially overlapping with those observed following IFN‐γ treatment and conferred neuroprotection, indicating that selective hemichannel inhibition can limit damaging signaling while preserving beneficial network coupling. These findings reinforce the concept that the duration and type of Cx43 modulation are critical determinants of outcome.

Thus, these convergent effects support a functional association between IFN‐γ signaling and Cx43 hemichannel activity, although the requirement of Cx43 for IFN‐γ‐induced protection remains to be directly established. This mechanism aligns with broader channel‐based paradigms in neuropathic pain, including ATP‐release pathways mediated by pannexin channels downstream of purinergic signalling [52, 53], highlighting convergent routes through which excitability and injury propagation can be regulated.

## 5 Conclusions and Future Perspectives

In conclusion, our findings demonstrate that oxaliplatin‐induced neurotoxicity, characterized by the upregulation of pro‐inflammatory and pro‐nociceptive factors and increased neuronal hyperexcitability—key contributors to neuropathic pain in vivo—can be mitigated by IFN‐γ in an organotypic spinal cord model. These findings provide a basis for future in vivo studies aimed at validating the neuroprotective actions of IFN‐γ and further elucidating the mechanisms involved in OIPN.

## Author Contributions

A.T.: data curation, formal analysis, methodology, writing – original draft. V.F.: data curation, formal analysis, methodology, writing – original draft. E.L.: conceptualization, visualization, writing and editing; L.M.: visualization, writing and editing; C.C.: formal analysis and methodology; F.M.: formal analysis and methodology; D.V.: formal analysis and methodology; C.P.: formal analysis and methodology. E.G.: formal analysis, methodology and writing. L.C.: formal analysis, methodology and writing – original draft. A.M.: conceptualization, supervision, writing and editing. T.M.: formal analysis and methodology. G.M.: conceptualization and supervision. C.G.: conceptualization, funding acquisition, supervision, writing and editing. L.D.C.M.: conceptualization, funding acquisition, supervision, writing and editing.

## Funding

This work was supported by KNOWPAIN, a grant funded by the Tuscany region to C.G., by the National Recovery and Resilience Plan (NRRP), project MNESYS (PE00000006)—A Multiscale integrated approach to the study of the nervous system in health and disease (DN. 1553 11.10.2022) to C.G. and by the Italian Ministry of University and Research (MIUR)—“Dipartimenti di Eccellenza 2023–2027”—58514_DIPECC_23_27—to the Department NEUROFARBA.

## Disclosure

The images shown in the figures panels to illustrate immunofluorescence data were processed using the Thunder algorithm (Leica Software) to reduce background noise. Entire Thunder‐processed images were subsequently adjusted for contrast and brightness to improve presentation clarity.

## Ethics Statement

Formal approval to conduct the study was obtained from the Italian Ministry of Health (No. 17.E9C.N.XCN), from the Animal Subjects Review Board of the University of Florence.

## Conflicts of Interest

The authors declare no conflicts of interest.

## Supporting information


**Figure S1:** Cytotoxicity assessment in organotypic spinal cord slices treated with IFN‐γ. LDH quantification in the culture medium after 6 (A) and 24 (B) hours of treatment with three different concentrations of IFN‐γ (6.5, 65, and 200 ng/mL). Results are expressed as mean ± SEM (*n* = 6) of three independent experiments. Values are expressed as a percentage of the control, arbitrarily set at 100.
**Figure S2:** Concentration of Substance P (A) and CGRP (B) determined in the media of organotypic spinal cord slices by ELISA. After 3 and 6 h of treatment with oxaliplatin (10 μM) and IFN‐γ (65 ng/mL), both as single treatments and in co‐treatment. Results are expressed as a percentage relative to the control, which was arbitrarily set at 100%. Data are presented as mean ± SEM of three independent experiments. *p < 0.005; **p < 0.01; ***p < 0.001 vs. control.
**Figure S3:** Analysis of passive properties of somatosensory neurons in the dorsal horn of organotypic spinal cord slices. (A) Membrane Capacitance (pF), (B) Membrane Resistance (MΩ), (C) Resting Membrane Potential (mV), and (D) Threshold (mV) measured after 24 h of treatment with oxaliplatin (OXA, 10 μM), IFN‐γ (65 ng/mL), or their combination. Gap19 (10 μM) was applied as a 30‐min pretreatment, either alone or together with oxaliplatin. Bars represent the mean ± SEM of at least 15 cells in three independent experiments. **p < 0.01 vs. control; ^^*p* < 0.01 vs. oxaliplatin.
**Figure S4:** Analysis of sEPSCs properties of somatosensory neurons in the dorsal horn of organotypic spinal cord slices. (A) Rise tau (ms), (B) Decay tau (ms), and (C) Area (ms*pA) measured after 24 h of treatment with oxaliplatin (OXA, 10 μM), IFN‐γ (65 ng/mL), or their combination. Gap19 (10 μM) was administered as a 30‐min pretreatment, either alone or together with OXA. Bars represent the mean ± SEM of at least 16 cells in three independent experiments.

## Data Availability

The data that support the findings of this study are available from the corresponding author upon reasonable request.
