# Supplementary material for: Interferon‐γ Mitigates Oxaliplatin‐Induced Neurotoxicity: Analogies With Connexin 43
Source: CNS Neurosci Ther. 2026 Oct 22;32(10):e71197. doi: 10.1002/cns.71197 (PMC13687928; doi:10.1002/cns.71197)
Supplement: Supplementary file 1 [file CNS-32-e71197-s001.docx]

**Supplementary material**

**Figure S1.** *Cytotoxicity assessment in organotypic spinal cord slices treated with IFN-γ.* LDH quantification in the culture medium after 6 (A) and 24 (B) hours of treatment with three different concentrations of IFN-γ (6.5, 65, and 200 ng/mL). Results are expressed as mean ± S.E.M (n=6) of three independent experiments. Values are expressed as a percentage of the control, arbitrarily set at 100

**Figure S2.** *Concentration of Substance P (A) and CGRP (B) determined in the media of organotypic spinal cord slices by ELISA.* After 3 and 6 hours of treatment with oxaliplatin (10 µM) and IFN-γ (65 ng/mL), both as single treatments and in co-treatment. Results are expressed as a percentage relative to the control, which was arbitrarily set at 100%. Data are presented as mean ± S.E.M of three independent experiments. *p < 0.005; **p < 0.01 vs control

**Figure S3.** *Analysis of passive properties of somatosensory neurons in the dorsal horn of organotypic spinal cord slices.* (A) Membrane Capacitance (pF), (B) Membrane Resistance (MΩ), (C) Resting Membrane Potential (mV), and (D) Threshold (mV) measured after 24 hours of treatment with oxaliplatin (OXA, 10 μM), IFN-γ (65 ng/mL), or their combination. Gap19 (10 μM) was applied as a 30-minute pretreatment, either alone or together with oxaliplatin. Bars represent the mean ± S.E.M. of at least 15 cells in three independent experiments. *p < 0.05; **p < 0.01 vs control; ^p < 0.05 vs oxaliplatin

**Figure S4.** *Analysis of sEPSCs properties of somatosensory neurons in the dorsal horn of organotypic spinal cord slices.* (A) Rise tau (ms), (B) Decay tau (ms), and (C) Area (ms*pA) measured after 24 hours of treatment with oxaliplatin (OXA, 10 μM), IFN-γ (65 ng/mL), or their combination. Gap19 (10 μM) was administered as a 30-minute pretreatment, either alone or together with OXA. Bars represent the mean ± S.E.M. of at least 16 cells in three independent experiments
